# Characterization and Therapeutic Potential of Curcumin-Loaded Cerium Oxide Nanoparticles for Interstitial Cystitis Management

**DOI:** 10.3390/antiox13070826

**Published:** 2024-07-10

**Authors:** Yang-Chen Lin, Ya-Jyun Liang, Chun-Hong Zhang, Li-Jia Liu, Feng-Huei Lin

**Affiliations:** 1 Department of Biomedical Engineering, College of Medicine and College of Engineering, National Taiwan University, Taipei 10617, Taiwan; leo90307@gmail.com (Y.-C.L.); d04548016@ntu.edu.tw (Y.-J.L.); 2 Yantai Research Institute, Harbin Engineering University, 145 Nantong Street, Nangang District, Harbin 150009, China; zhangchunhong97@163.com (C.-H.Z.); liulijia@hrbeu.edn.cn (L.-J.L.); 3 Institute of Biomedical Engineering and Nanomedicine, National Health Research Institutes, Miaoli 35053, Taiwan

**Keywords:** interstitial cystitis, inflammation, reactive oxygen species, antioxidants, drug delivery, nanoparticles

## Abstract

Oxidative stress resulting from reactive oxygen species (ROS) is often considered to be the leading cause of interstitial cystitis (IC), which is a chronic inflammatory disease. Antioxidants have been proven to have promising therapeutic effects on IC. In this study, we present an antioxidant intervention for IC by introducing curcumin-loaded cerium oxide nanoparticles (Cur-CONPs). Recognizing oxidative stress as the primary contributor to IC, our research builds on previous work utilizing cerium oxide nanoparticles (CONPs) for their outstanding antioxidant and anti-inflammatory properties. However, given the need to effectively relieve acute inflammation, we engineered Cur-CONPs to harness the short-term radical-scavenging antioxidant prowess of curcumin. Through in vitro studies, we demonstrate that the Cur-CONPs exhibit not only robust antioxidant capabilities but also superior anti-inflammatory properties over CONPs alone. Furthermore, in vivo studies validate the therapeutic effects of Cur-CONPs on IC. Mice with IC subjected to the Cur-CONP treatment exhibited improved micturition behaviors, relief from pelvic pain sensitivity, and reduced expression of inflammatory proteins (*IL-6*, *IL-1β*, *TNF-α*, *Cox2*). These findings suggest that the synergistic antioxidant properties of the Cur-CONPs that combine the sustained antioxidant properties of CONPs and acute anti-inflammatory capabilities of curcumin hold promise as a novel treatment strategy for IC.

## 1. Introduction

Interstitial cystitis (IC) is a chronic and severely debilitating disease of the urinary bladder. It is characterized by excessive urgency and frequency of urination, suprapubic pain, dyspareunia, chronic pelvic pain, and negative urine cultures, for which conditions such as bacterial cystitis, urethritis, neoplasia, vaginitis, and vulvar vestibulitis should be ruled out. The disease progression is usually marked by flare-ups and recurrences [1,2,3,4]. Generally, the first line of treatment for IC involves oral administration of steroids, pentosan polysulfate sodium, antihistamines, and tricyclic antidepressants. If these oral measures are unresponsive, then bladder instillation by hydrodistension, dimethyl sulfoxide, heparin, or a combination of agents is applied to the patients. If the symptoms cannot be effectively relieved through such approaches, surgery may be the last choice for IC patients, which would cause drastic pain. It should be noted that all of the treatments mentioned above can only relieve the symptoms partially rather than cure the disease [5,6,7,8,9].

Many pathological mechanisms are still considered in the etiology; however, there is evidence that the dysfunctions may be a result of oxidative stress from damage to the bladder [10,11,12,13]. Free radicals or reactive oxygen species (ROS) may be produced continuously because of decreased blood flow, ischemia, hypoxia, and chronic inflammation. Harmful oxidative reactions occurring during these processes are often counteracted or lessened by enzymatic and nonenzymatic antioxidant mechanisms. If the production of ROS is not balanced by scavenging via the antioxidant mechanism, then the excess ROS may result in oxidative stress. When the excess ROS cannot be scavenged effectively, the oxidative stress induces a bladder barrier dysfunction that causes the urinary solutes to invade the underlying tissues. The invading solutes, such as potassium, would then cause inflammatory responses, sensory nerve hyperactivity, and IC [6,13,14]. If the ROS can be continuously scavenged from the inflammation site during the early or even later stages of IC, then the disease may be cured.

Cerium oxide has been shown to have similar antioxidant properties to superoxide dismutase and catalase in the diffusion of free radicals from the inflammation site [15]. Many studies have also shown that cerium ions may switch between their Ce^3+^ and Ce^4+^ states, accompanied by the production of oxygen vacancies inside the lattice. In the equilibrium state, a small quantity of Ce^3+^ occupies the Ce^4+^ positions in the crystal lattice, which may dynamically convert to the ground state to release one electron for the oxidation reaction. Once the Ce^3+^ concentration in the lattice is not maintained in the equilibrium condition, Ce^4+^ is switched back to Ce^3+^ as a sink for free electrons to complete the reduction reaction [16,17], thereby allowing constant diffusion of ROS and relief from IC symptoms for a longer duration. Previously, cerium oxide nanoparticles (CONPs) have been prepared to ensure a large surface area because of their high surface-to-volume ratio for purging ROS in IC [18]; although this produced therapeutic effects, it did not provide the desired relief from acute pain.

To address this limitation, we attempted to use an antioxidant to relieve the acute pain experienced by IC patients. Curcumin, a chemical derived from turmeric, has solid antioxidant and anti-inflammatory properties [19]; the ROS-scavenging properties of curcumin are owed to its hydroxy phenolic groups. Curcumin presents short-term antioxidant properties that can improve acute inflammatory responses [20]; however, curcumin shows limited therapeutic effects when used orally owing to its poor bioavailability and instability within the gastrointestinal (GI) tract and physiological environment [21]. Despite its known limitations in bioavailability and mixed results in clinical trials, curcumin was selected for this study due to its well-documented antioxidant and anti-inflammatory properties. In the present study, we used homemade CONPs as the carrier to load curcumin for treating acute pain caused by IC, and the CONPs were expected to play a role in the long-term diffusion of oxidative stress as noted in previous studies. This study thus aims to evaluate the therapeutic effects of curcumin-loaded cerium oxide nanoparticles (Cur-CONPs) on IC.

**Figure 1:**
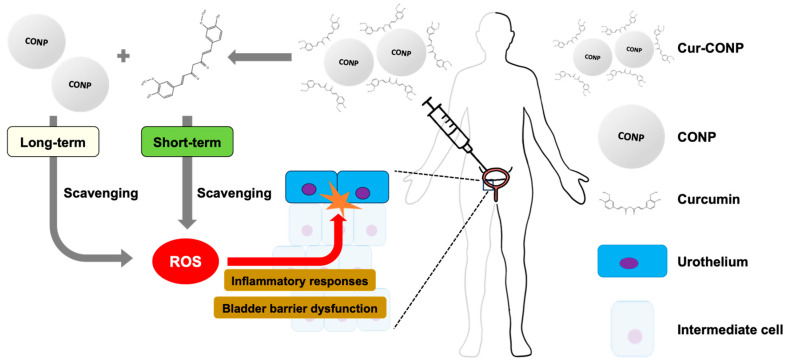
A brief illustration of the study procedures. After administration, curcumin is quickly released, serving as a short-term antioxidant for acute pain. CONPs serve as long-term antioxidants. By synergically scavenging ROS, which causes bladder barrier dysfunction and inflammatory responses, Cur-CONPs can effectively improve symptoms of IC.

The overall concept behind solving the problems of IC patients is shown in Figure 1.

## 2. Materials and Methods

### 2.1. Synthesis of CONPs

The CONPs were synthesized using the homogeneous precipitation method, which is a lab-designed, eco-friendly process modified from a traditional method. In short, 0.0375 M Ce(NO_3_)_3_∙6H_2_O (99.5%, Alfa Aesar, Waltham, MA, USA) solution and an equal volume of 0.5 M hexamethylenetetramine (HMTA, 99.5%, Alfa Aesar, USA) solution were stirred at room temperature for one day. The mixture was then centrifuged at 10,000 rpm for 10 min. The precipitate was collected and washed twice with dd-H_2_O and 99% ethanol alternately to obtain the CONPs. This method produces CONPs with good crystal and uniformly nanosized structures at room temperature and ambient pressure. 

### 2.2. Synthesis of Cur-CONPs

The process of synthesizing the Cur-CONPs is briefly described as follows: Firstly, 15 mL of 8 mg/mL curcumin (Sigma, St. Louis, MO, USA) in acetone was mixed with 240 mL of 0.7 mg/mL CONPs in dd-H_2_O. The mixture was stirred in the dark overnight and then centrifuged at 10,000 rpm for 10 min. Lastly, the precipitate was collected to obtain Cur-CONPs after washing thrice with dd-H_2_O.

### 2.3. Material Characterizations of the CONPs and Cur-CONPs

The synthesized CONP and Cur-CONP powders were ground and passed through a 200-mesh sieve. The samples were then mounted in glass holders with a slim ditch in the central area. The XRD patterns were obtained using an X-ray diffractometer (Rigaku, TTRAX III, Tokyo, Japan) equipped with a Cu-Kα rotation anode with operation at a voltage of 40 kV and a current of 20 mA. The scan rate was 0.025 degree/step at 5 s/step, and the 2-theta range was from 10° to 80°. The data were analyzed using MDI JADE 6.5 software to identify the crystal structures. 

The CONP and Cur-CONP powders were prepared similarly to those used for XRD. The sample powders were mixed with potassium bromide in a ratio of 1:100 and pressed into disks for FTIR analyses. The FTIR spectra (Spotlight 200i Sp2 with AutoATR System, Waltham, MA, USA) were analyzed for the functional groups of the synthesized materials in the scanning range of 500–4000 cm^−1^ [22].

The morphologies and particle sizes of the CONPs and Cur-CONPs were examined using an SEM (Hitachi S-4800 field-emission scanning electron microscope with energy-dispersive X-ray spectrometer, QUANTAX Annular XFlash^®^ QUAD FQ5060, Tokyo, Japan). The sample powders were dispersed on sample stages and coated with thin Pt films via sputtering physical vapor deposition (PVD) to diffuse the edge discharge. The applied voltage for SEM was 10 kV. 

The electron diffraction patterns and lattice images were obtained via a field-emission TEM (JEOL 2010F, Tokyo, Japan) to check the crystal structures; the accelerating voltage used was 200 kV. The sample powders were spread on carbon-coated Ni grids and then mounted on holders. The TEM device was equipped with an energy-dispersive X-ray (EDX) system, which was used to analyze the chemical composition.

The hydrodynamic diameters and zeta potentials were analyzed using a Zetasizer Nano (Malvern Instruments, Zetasizer NanoZS, Westborough, MA, USA) based on the DLS method. The hydrodynamic diameter can be expressed in terms of the particle size. The chemical states of Ce in the CONPs and Cur-CONPs were studied via XPS (ESCA0038, ULVAC-PHI, Tokyo, Japan).

### 2.4. Curcumin Loading Efficiency and Loading Capacity

The curcumin loading in the Cur-CONPs was analyzed with UV-vis light by interpolating the calibration curve prepared using standardized curcumin concentrations. About 1 mg of Cur-CONP was placed in 50 mL of 99% ethanol. The absorbance values were measured at 425 nm.

### 2.5. In Vitro Curcumin Release Profile of Cur-CONPs

To study the release profile of curcumin, 1 mg of Cur-CONPs was immersed in a 50 mL phosphate-buffered saline (PBS, 1X, pH 7.4) and maintained at a steady state for different time periods of 20 min, 40 min, 1 h, 1.5 h, 8 h, 24 h, 48 h, and 96 h. The immersed samples at different time periods were centrifuged at 10,000 rpm for 10 min. Then, about 1 mL of the suspension was retrieved to measure the curcumin loading by UV-vis at 425 nm. 

### 2.6. Cell Culture

Human bladder carcinoma cells (T24, BCRC, 60062, Hsinchu, Taiwan) were cultured in McCoy’s 5a medium (Sigma, USA) supplemented with 1.5 mL of L-glutamine, a 10% (*v*/*v*) fetal bovine serum (Gibco, Thermo Fisher Scientific, Waltham, MA, USA), and a 1% (*v*/*v*) penicillin/streptomycin. The T24 cells were cultured in a humidified incubator (Thermo, FV300, Waltham, MA, USA) set to 37 °C and 5% CO_2_.

### 2.7. In Vitro Biocompatibility

The cell viabilities and cytotoxicities were evaluated using a WST-1 assay and a Live/Dead stain on the T24 cells, respectively. The experiments were divided into five groups: the Control group contained cultured T24 cells on the culture dish without any treatment; the Positive group had an added 0.2 mg/mL zinc diethyldithiocarbamate (ZDEC, Sigma, USA) or a 0.1% Triton X-100 (Sigma, USA) as a toxic substance in the culture containing the T24 cells; the Negative group had 0.2 mg/mL of nontoxic aluminum oxide (Al_2_O_3_, Sigma, USA) added to the T24 cells; the CONP group contained T24 cells cultured with 0.2 mg/mL of CONPs; the Cur-CONP group contained T24 cells cultured with 0.2 mg/mL of Cur-CONPs.

For the cell viability test, the T24 cells were seeded in a 96-well culture dish at a density of 10^4^ cells/well and cultured for one day. Subsequently, the medium was replaced with a culture medium or sample-extracting medium and cultured for one more day. The WST-1 reagent (Takara, Berkeley, CA, USA) at 10% (*v*/*v*) was then added, and the sample was incubated in the dark for 2 h. The medium was harvested and then analyzed by an ELISA Reader at 450 nm to obtain the optical density (OD) values.

For the cytotoxicity test, the T24 cells were seeded in a 3 cm culture dish at a density of 1.5 × 10^5^ and cultured for one day. The medium was then replaced with the culture medium or sample-extracting medium and cultured for one more day. The Live/Dead reagent (2 μM calcein AM and 4 μM EthD-1 solution) was added next and reacted for 40 min. The results were examined under a fluorescent microscope (Olympus, BX 51, Tokyo, Japan), where the living cells were marked in green and dead cells were marked in red. 

### 2.8. In Vitro Study: Evaluation of Intracellular ROS

To evaluate the antioxidant properties, the intracellular ROS were measured using a DCFDA assay, where the T24 cells were seeded in a 6-well plate. The in vitro oxidative stress model was established by adding 4-hydroperoxy cyclophosphamide (4-HC, USBiological, Salem, MA, USA); 4-HC is an analog of cyclophosphamide that is converted to acrolein to induce oxidative stress that damages the surrounding tissues. These results could be considered as reference doses for in vivo IC models. 

The experiments consisted of four distinct groups: Control, 4-HC, CONP, and Cur-CONP. The Control group included cells without additional treatments. The 4-HC group included cells treated with 4-HC. The CONP group included cells treated with 10 μg CONPs, and the Cur-CONP group included cells treated with 10 μg Cur-CONPs. To induce oxidative stress in the cells, the experimental samples, except for the Control group, were treated with 37.5 μM 4-HC and reacted for 4 h before being washed with PBS. Thereafter, 25 μM of the DCFDA reagent was added to each sample and incubated at room temperature for 45 min. The results were then recorded and evaluated using a fluorescence microscope (Olympus, BX 51, Japan).

### 2.9. In Vitro Study: Anti-Inflammatory Abilities of the Materials

To validate the anti-inflammatory abilities of the synthesized materials, their pro-inflammatory gene expressions were measured via RT-qPCR. The experimental groups were divided as follows: Control, 4-HC, CONP, and Cur-CONP. The procedure for each experimental group followed similar steps as described in the section on evaluating intracellular ROS. The only difference here was to isolate the RNA after 4 h of treatment with 4-HC. In short, the RNA was isolated with TRI reagent (Sigma, USA) and Direct-zolTM RNA MiniPrep Kits (Zymo Research, Solana Beach, CA, USA). After extracting the RNA, complementary DNA (cDNA) synthesis was carried out using SuperScript TM IV Reverse Transcriptase (Thermo Fisher, Waltham, MA, USA) according to the manufacturer’s instructions. The qPCR was performed with SYBR Green Master Mix (Thermo Fisher, USA), and the results were evaluated using a LightCycler^®^ 480 Instrument (Roche Diagnostics Nederland BV, Almere, The Netherlands). The relative gene expressions were interpreted in terms of 2^−ΔΔCt^. The sequences of the primers are listed in Table 1.

### 2.10. Animal Study

The animal studies used herein were approved by the Institutional Animal Care and Use Committee of National Taiwan University as per established protocols. Eight-week-old ICR mice were housed in a light-controlled room (12 h light/dark cycle) with adequate humidity and temperature. Rodent food and water were provided ad libitum. After the mice had adapted to the cage environment for 2 weeks, the 10-week-old ICR mice were used in the animal studies. 

The adapted ICR mice were administered intraperitoneal (IP) injections of cyclophosphamide (CYP) to induce IC, and the CYP would be later metabolized to acrolein in the liver [23]. Acrolein is a toxic substance that causes oxidative stress and damages the surrounding tissues [24]. The animal model was used to study the therapeutic effects of the Cur-CONPs on IC.

### 2.11. In Vivo Experiment Design

The experiments were divided into four groups: Control, CYP, CONP, and Cur-CONP. In the Control group, the mice were administered PBS by IP injection on days 0, 4, 7, 9, 11, and 13. The mice in the CYP group were treated with CYP at a rate of 80 mg/kg by IP injection on days 7, 9, 11, and 13 to induce IC. In the CONP group, the mice were administered 80 mg/kg of CYP by IP injection on days 7, 9, 11, and 13, along with 30 mg/kg of CONPs on days 0 and 4. For the Cur-CONP group, the mice were treated with 80 mg/kg of CYP by IP injection on days 7, 9, 11, and 13, along with 30 mg/kg of Cur-CONPs on days 0 and 4. All mice were weighed before the injections. The experimental ICR mice were then subjected to several tests, as noted below, before being euthanized. The duration of the experiment was 14 days.

### 2.12. Micturition Behavior Study

The micturition behaviors of the mice were evaluated using the Void Spot assay, where the mice were placed individually in a mesh-bottomed cage and elevated on a wire grid above filter paper (Whatman No.1, AW1001-00917, Shrewsbury, MA, USA) for 3 h [25]. To prevent misleading results, the mice were restricted food and water during the Void Spot assay. After collection and drying at room temperature, the filter papers were analyzed and recorded using the Alpha Innotech FluorChem SP Digital Imaging System (Alpha Innotech, San Leandro, CA, USA).

The numbers of void spots and total areas of the void spots were related to the frequency and urinary volumes of the mice, respectively, which could be directly translated to their micturition behaviors under IC. This study examined both the total void volume and the void spot number for several reasons. First, this dual approach allowed a more nuanced understanding of the urinary dynamics under investigation. Assessing the total void volume allowed capturing the overall quantitative aspects of the voiding behaviors. Simultaneously, analyzing the void spot numbers could provide valuable insights into the frequency, offering more detailed perspectives on the urinary patterns. This combined approach enables holistic exploration of the factors influencing urinary function and contributes to a more thorough interpretation of the experimental outcomes.

### 2.13. Pelvic Pain Evaluation

The pelvic pain of the mice was evaluated using the Von Frey test. The mice were placed on top of a wire floor grid (Stoelting Co., Wood Dale, IL, USA) for 15 min until they were acclimatized. The withdrawal responses of the mice were then observed and recorded after stimulation via the Von Frey filament. Different force filaments of 0.8, 1.8, 2.9, 11, 15, 32, and 74 g-force were applied to the lower abdomen five times in ascending order. Immediate licking or scratching of the filament stimulation site, sharp retraction of the abdomen, and jumping were considered as the withdrawal responses [26,27]. The measurement of the response rate in the Von Frey test serves as an indicator of the overall sensitivities of the mice to mechanical stimuli, offering qualitative insights into their nociceptive responses and the potential presence of hyperalgesia.

The mice were also tested for mechanical withdrawal thresholds via the “ascending stimulus” method. Von Frey filaments were sequentially applied manually to the lower abdomen area in order of increasing force, beginning with the lowest force (0.8 g-force). Each filament was applied five times, and the mechanical withdrawal threshold was determined as the force at which a positive withdrawal response was 40% or more (i.e., withdrawal response is elicited in two or more out of five applications) [28]. The measurement of the mechanical withdrawal threshold in the Von Frey test aims to quantify the sensitivity of the mice to mechanical stimuli, offering a precise numerical representation of tactile allodynia. A lower withdrawal threshold suggests increased sensitivity, providing quantitative information on the nociceptive processing.

### 2.14. Histological Evaluation of the Bladder

After the experimental mice were euthanized, their bladders were harvested and washed with a 10% formalin solution. Each bladder was halved and then immersed in 10% formalin for 24 h [29]. The samples were cleaned using PBS and dehydrated with a series of alcohols at concentrations of 75%, 80%, 90, 95%, and 100% before being placed into tissue boxes embedded in parafilm blocks [30]. Each embedded tissue block was sectioned into thin films of 5 μm thickness and H&E stained. The hematoxylin dye stained the nucleus to a blue-purple color, while the eosin dye stained the cytoplasm to a pink color; these allowed for examination of the microstructure of the bladder wall and urothelium layer of the bladder under an optical microscope.

The surface structures of the bladder wall and urothelium arrangement were further examined via SEM. The harvested bladder samples were fixed in 10% glutaraldehyde and washed with PBS. The samples were dehydrated with a series of alcohols and further dried in liquid CO_2_ using a critical point dryer to prevent tissue collapsing [31]. The dried samples were coated with a Pt-thin film by sputtering PVD.

The bladder microstructures were more closely examined at the cellular level via the TEM. The bladder was harvested and cut into a block of 0.5 mm^3^ immediately after the mice were euthanized [32]. The sample blocks were fixed in 2.5% glutaraldehyde in 0.1 M PBS for 1 h and then immersed in 1% osmium tetroxide in 0.1 M PBS for 1 h. After fixation and OsO4 staining, the blocks were dehydrated in different concentrations of ethanol from 35% to 100% and embedded in Spur’s low-viscosity resin. Ultrathin sections were prepared using a Leica EM UC7 Ultramicrotome (Leica Microsystems, Wetzlar, Germany) and stained with uranyl acetate and lead citrate. The sections were observed via the TEM (JEOL JEM-1400, Peabody, MA, USA) at an acceleration voltage of 80 kV.

### 2.15. Western Blot Analysis

The anti-inflammatory abilities of the developed materials were evaluated by Western blotting. The samples were briefly prepared as follows: Each harvested bladder was mechanically homogenized and centrifuged. The suspension was then collected and immersed in RIPA buffer before being stored at −80 °C until use. Protein concentrations were measured by the Bio-Rad Protein Assay (Bio-Rad, Hercules, CA, USA), where the samples retrieved from the −80 °C storage were supplemented with a 2-mercaptoethanol (β-ME, Bio-Rad, USA), protease inhibitor cocktail (MedChemExpress, Monmouth Junction, NJ, USA), and a phosphatase inhibitor cocktail (Calbiochem, San Diego, CA, USA). To denature the proteins, the samples were boiled for 5 min at 95 °C before electrophoresis. 

The proteins were separated using SDS-polyacrylamide gel electrophoresis (SDS-PAGE). A 30 μg sample and a marker (PM2500, SMOBIO Technology, Paramount, CA, USA) were loaded on the lane of a 10% acrylamide supporting gel. Initially, the samples underwent electrophoresis at 60 V for 20 min and at 140 V until the dye crossed over the length of the gel. Subsequently, protein transfer was performed on a polyvinylidene difluoride (PVDF, Cytiva, Dreieich, Germany) membrane at 4 °C and a transfer condition at 300 mA for 90 min. The membranes were then blocked with the TOOLSpeed Blocking Reagent (TFU-IHC01, BIOTOOLS, New Taipei City, Taiwan) for 90 s and rinsed with tris-buffered saline containing 0.1% Tween^®^ 20 detergent (TBST). The membranes were individually incubated with the following primary antibodies overnight at 4 °C: rabbit anti-β-actin (1:1000, 4970, Cell Signaling Technology, Danvers, MA, USA), rabbit anti-IL-6 (1:1000, 12912S, Cell Signaling Technology, USA), rabbit anti-IL-1β (1:2000, 26048-1-AP, Cell Signaling Technology, USA), rabbit anti-TNF-α (1:1000, 17590-1-AP, Cell Signaling Technology, USA), and rabbit anti-COX-2 (1:200, 160107, Cell Signaling Technology, USA). After rinsing with TBST overnight, the membranes were again incubated with goat anti-rabbit IgG (1:5000, GTX213110-01, Cell Signaling Technology, USA) at room temperature for 1 h. Finally, the chemiluminescent horseradish peroxide (HRP) substrate (Immobilon^®^ Western WBKLS0500, Merck, Darmstadt, Germany) was used to detect the immunocomplexes. The results were analyzed using Image Lab Software version 5.2.

### 2.16. Statistical Analysis

The experimental data were expressed as the mean ± standard deviation (SD). Different treatments were compared based on statistical significance and analyzed by one-way ANOVA. *p* < 0.05 was considered to be statistically significant. The statistical significances used were as follows: * *p* < 0.05, ** *p* < 0.01, *** *p* < 0.001, and **** *p* < 0.0001.

## 3. Results

### 3.1. Material Characterizations

**Figure 2:**
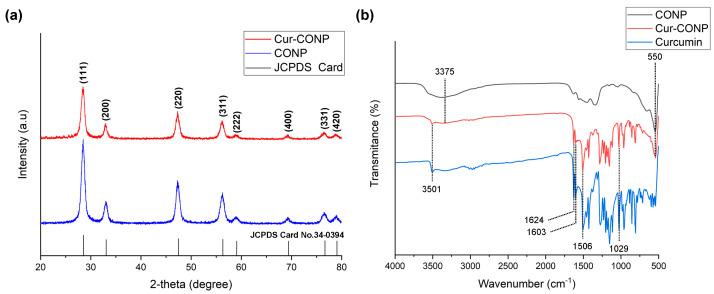
(**a**) XRD patterns showing the (111), (200), (220), (311), (222), (400), (331), and (420) planes of CONP and Cur-CONP. Matching with JCPDS Card No.34-0394 confirms cerium oxide crystallinity. Shared Ce–O band indicates CONP presence in Cur-CONP. Curcumin incorporation does not affect cerium oxide crystallinity, ensuring successful synthesis. (**b**) Fourier transform infrared (FTIR) spectra illustrating the functional groups of CONPs, Cur-CONPs, and curcumin. Cur-CONPs present the functional groups of both CONPs and curcumin. Peaks corresponding to –OH stretching, Ce–O stretching, and phenolic –OH bond are observed at 3375 cm^−1^, 550 cm^−1^, and 3501 cm^−1^, respectively. Distinctive absorption peaks at 1624 cm^−1^ and 1603 cm^−1^ indicate the presence of the carbonyl group, while C–O–C stretching at 1029 cm^−1^ and ethylene group at 1506 cm^−1^ confirm the incorporation of curcumin in Cur-CONP. The FTIR spectrum of Cur-CONP demonstrates absorption peaks characteristic to both CONPs and curcumin, validating the successful synthesis of Cur-CONPs.

The XRD patterns of the CONPs and Cur-CONPs exhibit prominent peaks at 28.56°, 33.08°, 47.47°, 56.36°, 59.08°, 69.40°, 76.70°, and 79.07° corresponding to the (111), (200), (220), (311), (222), (400), (331), and (420) lattice planes, respectively (Figure 2a). A comparison of these patterns with JCPDS Card No.34-0394 revealed a consistent match, affirming the presence of well-defined peaks and robust crystallinity of cerium oxide in both the CONPs and Cur-CONPs. Notably, the incorporation of curcumin did not interfere significantly with the crystalline structure of cerium oxide, as evidenced by the maintained clarity and distinctiveness of the XRD peaks. These results underscore the compatibility of curcumin loading with the inherent crystalline integrity of cerium oxide in the synthesized material.

The functional groups of the CONPs, Cur-CONPs, and curcumin were characterized by FTIR spectra, as shown in Figure 2b. The broad peak observed at 3375 cm^−1^ corresponds to –OH stretching, while the peak at 550 cm^−1^ signifies Ce–O stretching [33]. The persistence of the Ce–O band in both the CONPs and Cur-CONPs underscores the inclusion of CONPs within the Cur-CONP construct. The peak at 3501 cm^−1^ is attributed to the phenolic –OH bond [34]. The distinct absorption peaks at 1624 cm^−1^ and 1603 cm^−1^ related to the carbonyl group, C–O–C stretching at 1029 cm^−1^, and the ethylene group at 1506 cm^−1^, confirm the presence of curcumin in the Cur-CONPs [35]. The FTIR spectrum of the Cur-CONPs exhibits absorption peaks characteristic of both CONPs and curcumin, providing conclusive evidence for the successful synthesis of Cur-CONPs.

**Figure 3:**
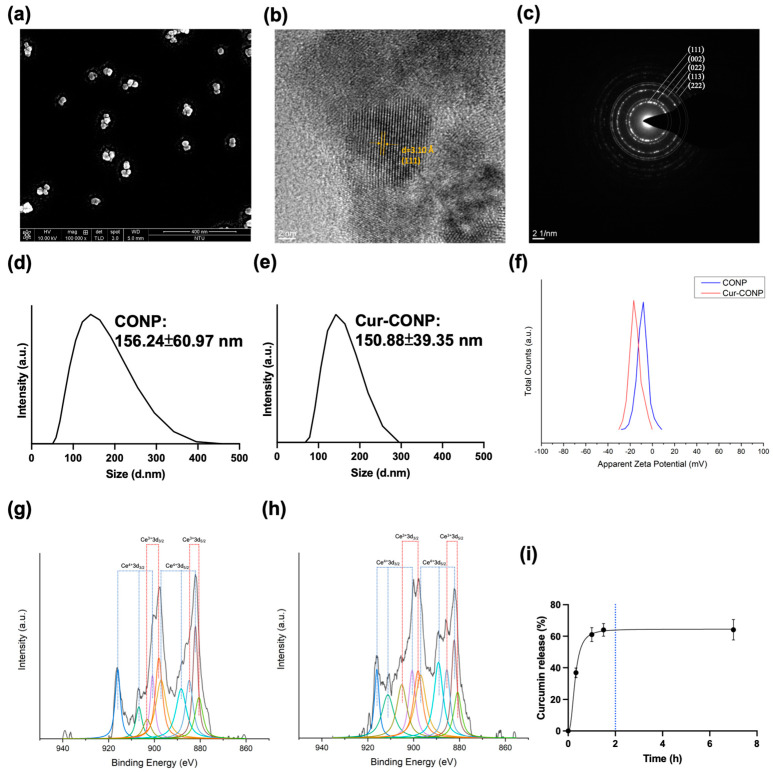
(**a**) An SEM image of Cur-CONP showing that the particles present no significant aggregation. (**b**) A high-resolution TEM image of Cur-CONPs showing the interplanar spacing of the lattice plane (111): scale bar, 2 nm. (**c**) The TEM electron diffraction pattern of Cur-CONPs showing the lattice planes (111), (002), (022), (113), and (222). The results correspond with those of the XRD pattern. The size distributions of (**d**) CONPs (156.24 ± 60.97 nm) and (**e**) Cur-CONPs (150.88 ± 39.35 nm) show a slight increase in the hydrodynamic diameter after curcumin loading. (**f**) The zeta potentials of the CONPs and Cur-CONPs (−8.91 ± 4.84 mV and −15.54 ± 4.96 mV, respectively) reveal that curcumin loading induced a more negative zeta potential for Cur-CONP, enhancing its colloidal stability. The XPS data of (**g**) CONPs and (**h**) Cur-CONPs show the chemical states of the elements. By calculating the area under the characteristic peaks, the Ce^3+^/Ce^4+^ ratios of CONP and Cur-CONP are 0.518 and 0.564, respectively, indicating enhanced antioxidant properties. The higher ratio attributed to a greater concentration of Ce^3+^ suggests increased oxygen vacancies in the crystal structure, potentially improving the oxygen exchange and redox reactions. (**i**) The drug release study indicated a rapid 60% release of curcumin within 2 h, aligning with enhancement of the short-term antioxidant properties to alleviate acute inflammation swiftly.

The SEM image (Figure 3a) shows well-dispersed particles, highlighting the colloidal stability of Cur-CONPs without significant aggregation. The lattice image of Cur-CONP with 3.10 Å of d-spacing was obtained under a high-resolution TEM (Figure 3b), consistent with the (111) plane and indicative of the fluorite structure of cerium oxide. The electron diffraction pattern (Figure 3c) aligns with the crystalline structure of cerium oxide, corroborating the XRD results and confirming the structural integrity of the Cur-CONPs.

The DLS analysis of particle size distributions of the CONPs and Cur-CONPs (Figure 3g) reveals hydrodynamic diameters of 156.24 ± 60.97 nm and 150.88 ± 39.35 nm, respectively. The slightly larger hydrodynamic diameter of the Cur-CONPs suggests a potential influence of the curcumin loading process. Notably, the distribution of Cur-CONPs exhibits a higher concentration than that of CONPs, underscoring its more focused and homogeneous presence in the solution. This concentration indicates a notable impact on the stability and uniform dispersion of Cur-CONPs, potentially enhancing their behavior in the solution phase. The zeta potential measurements for the CONPs and Cur-CONPs reveal values of −8.91 ± 4.84 mV and −15.54 ± 4.96 mV, respectively (Figure 3h). The larger magnitude of the zeta potential for the Cur-CONPs indicates increased electrostatic repulsion forces between the particles. This finding highlights the enhanced colloidal stability and more homogeneous distribution of the Cur-CONPs, aligning with the concentrated distribution observed for the hydrodynamic diameter of the Cur-CONPs. The elevated zeta potential of the Cur-CONPs suggests improved stability and uniform dispersion in solution, indicating favorable characteristics for potential applications.

To examine the chemical state and estimate the ratio of Ce^3+^/Ce^4+^, the CONPs and Cur-CONPs were analyzed using XPS; these spectra are shown in Figure 3g,h. The results show peaks for Ce^3+^3/2, Ce^3+^5/2, Ce^4+^3/2, and Ce^4+^5/2 [36,37]. The XPS peaks are listed in Table S1. The ratio of Ce^3+^/Ce^4+^ was calculated using the following equation:

Ratio of Ce^3+^/Ce^4+^ (%) = Area_Ce3+_/Area_Ce4+_ × 100% (1)

The areas under the characteristic peaks of Ce^3+^ and Ce^4+^ were denoted as Area_Ce3+_ and Area_Ce4+_, respectively. Unexpectedly, quantitative analysis revealed a Ce^3+^/Ce^4+^ ratio of 0.518 for the CONPs and 0.564 for the Cur-CONPs. A higher Ce^3+^/Ce^4+^ ratio indicates enhanced antioxidant properties, as a greater concentration of Ce^3+^ signifies increased oxygen vacancies in the crystal structure, which facilitate oxygen exchange and redox reactions [38,39]. This suggests that the complexation with curcumin elevates the antioxidant capacity of the CONPs, potentially augmenting therapeutic efficacy in individuals with IC.

In the investigation of drug loading capacity, the Cur-CONPs exhibit a drug loading capacity of 26.73% and a drug loading efficacy of 69.12%, signifying substantial loading content. The outcomes of the drug release study, illustrated in Figure 3i, reveal a rapid release profile, with 60% of the curcumin released within 2 h. This swift-release pattern aligns with the intention to augment the short-term antioxidant properties for mitigating acute inflammation by introducing curcumin. Following administration, the prompt release of curcumin enables rapid scavenging of the surrounding excess ROS.

### 3.2. In Vitro Study: Validation of Antioxidant and Anti-Inflammatory Properties of Cur-CONPs

**Figure 4:**
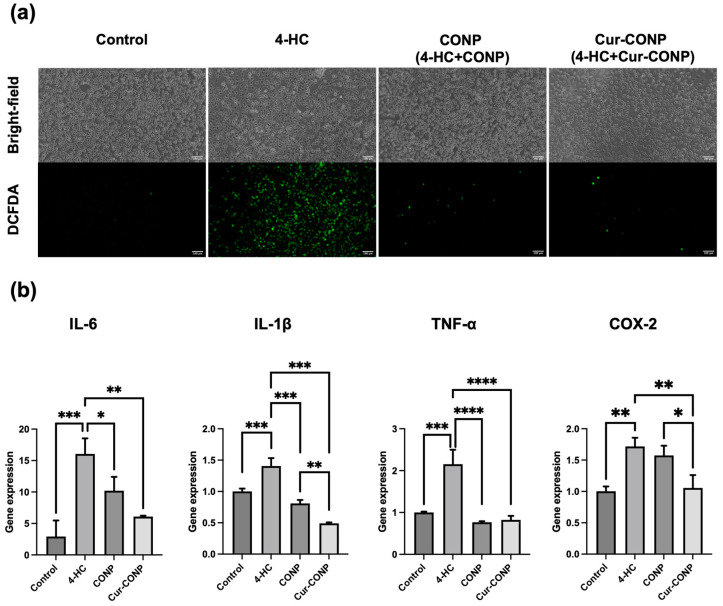
(**a**) The DCFDA assay results illustrating intracellular ROS levels. The lower fluorescence of the CONP and Cur-CONP groups than the 4-HC group validates the antioxidant properties of CONPs and Cur-CONPs, indicating their capability to scavenge intracellular ROS induced by 4-HC. Scale bars are 100 μm. (**b**) Expression levels of pro-inflammatory genes assessed by RT-qPCR. The 4-HC group shows the highest gene expression, while both CONP and Cur-CONP groups exhibit significantly lower levels, confirming their anti-inflammatory properties. Notably, Cur-CONP demonstrates superior anti-inflammatory efficacy to CONP, as indicated by the lower expression levels of *IL-1β* and *COX-2*. Control group: cells cultured in a medium without additional treatment; 4-HC group: cells treated with 4-HC; CONP group: cells treated with 4-HC and CONPs; Cur-CONP group: cells treated with 4-HC and Cur-CONPs. * *p* < 0.05, ** *p* < 0.01, *** *p* < 0.001, and **** *p* < 0.0001.

In the assessment of the antioxidant properties using the DCFDA assay, 2′,7′-dichlorofluorescein diacetate reacted with the intracellular ROS after penetrating the cell membranes. The subsequent transformation to 2′,7′-dichlorofluorescein (DCF) was detectable with an ELISA Reader by employing excitation and emission wavelengths of 485 nm and 535 nm, respectively. Consequently, a higher fluorescence intensity indicates elevated intracellular ROS levels. The results depicted in Figure 4a demonstrate that both the CONP and Cur-CONP groups exhibit lower fluorescence than the 4-HC group, which is treated with only 4-HC. These findings substantiate the antioxidant properties of the CONPs and Cur-CONPs, indicating their capacity to neutralize intracellular ROS induced by 4-HC.

The evaluation of the anti-inflammatory properties via RT-qPCR reveals that the 4-HC group exhibits the highest expression of pro-inflammatory genes. In contrast, the CONP and Cur-CONP groups display significantly lower expression levels, indicating that the expressions of the pro-inflammation genes are downregulated (Figure 4b). These results imply that the CONPs and Cur-CONPs have good anti-inflammatory properties. Additionally, in terms of *IL-1β* and *COX-2* expressions, the Cur-CONP group demonstrates lower pro-inflammatory gene expressions than CONP, suggesting superior anti-inflammatory properties of the Cur-CONPs over CONPs.

### 3.3. In Vivo Study: Evaluation of the Therapeutic Effects of Cur-CONPs through Micturition Behavior and Pelvic Pain

As mentioned, the CONP group included mice treated with cyclophosphamide and 30 mg/kg CONPs, while the Cur-CONP group included mice treated with cyclophosphamide and 30 mg/kg Cur-CONPs. Given that the loading capacity of curcumin is 26.73%, the CONP group received 30 mg/kg of CONPs, whereas the Cur-CONP group received 22 mg/kg of CONPs along with 8 mg/kg of curcumin.

**Figure 5:**
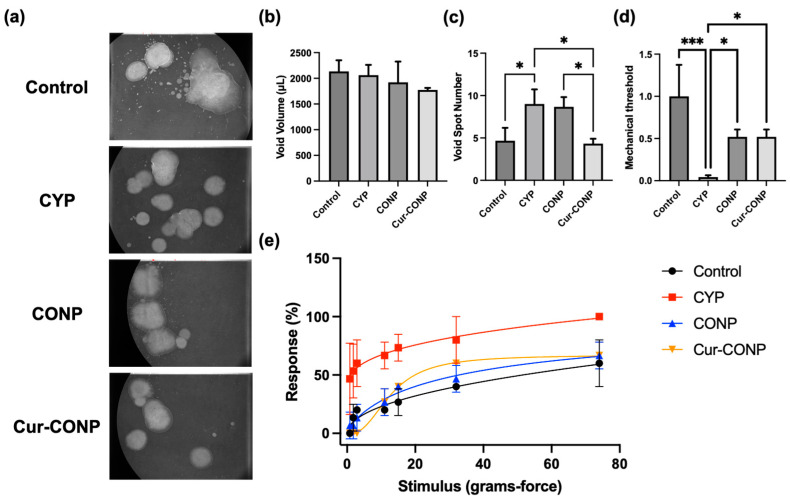
(**a**) Representative images after collecting and drying the filter papers of each group. (**b**) Total void volume measurements show no significant differences among the groups, indicating minimal influence on the subsequent urinary frequency study. (**c**) Urinary frequency results: CYP group mice exhibit the highest void spot number, indicating increased urinary frequency. In contrast, Cur-CONP group mice display significantly reduced urinary frequency compared to the CYP group. (**d**) Mechanical withdrawal threshold reveals heightened pain sensitivity in the CYP group and reduced sensitivity in the CONP and Cur-CONP groups. * *p* < 0.05 and *** *p* < 0.001. (**e**) Response rates confirm higher values in the CYP group and lower values in the CONP and Cur-CONP groups, supporting efficacy in alleviating pelvic pain in mice with IC. Control group: mice were administered sterile PBS; CYP group: mice were administered CYP; CONP group: mice were administered CYP and CONPs; Cur-CONP group: mice were administered CYP and Cur-CONPs.

In the micturition study, analysis of the filter papers was performed after collection and drying. Representative images of each group’s filter papers are illustrated in Figure 5a. The total void volume measurements revealed no significant differences among the experimental groups (Figure 5b). We believe that the void volume did not likely influence the subsequent urinary frequency study. In the urinary frequency study, a greater void spot number on the filter paper signified increased urinary frequency. These results reveal that the mice in the CYP group exhibited the highest number of void spots, indicating the highest urinary frequency. Conversely, mice in the Cur-CONP group demonstrated significantly reduced urinary frequency compared to the CYP group (Figure 5c). This observation demonstrates that Cur-CONPs can decrease urinary frequency in mice treated with CYP.

In assessing the therapeutic impact on pelvic pain in the mice, the Von Frey test entailed abdominal stimulation with various force filaments. Withdrawal responses were systematically gathered to quantify the mechanical thresholds and response rates as indicators of pain sensitivity. The mechanical threshold gauged the force required to elicit a withdrawal response; these results (Figure 5d) reveal that mice in the CYP group exhibit lower thresholds, suggesting heightened pain sensitivity compared to other groups. Conversely, the CONP and Cur-CONP groups displayed higher mechanical thresholds, indicating reduced pain sensitivities. This implies that Cur-CONPs may alleviate the pain sensitivity associated with IC. In addition to the withdrawal threshold, the study of response rate offers insights into the frequency of responses. The results show that the CYP group displayed higher rates, while the CONP and Cur-CONP groups exhibited lower rates (Figure 5e). These combined results suggest that both CONPs and Cur-CONPs can effectively mitigate pelvic pain induced by CYP in the mouse model of IC.

### 3.4. Histological Evaluation of the Mice Bladders

**Figure 6:**
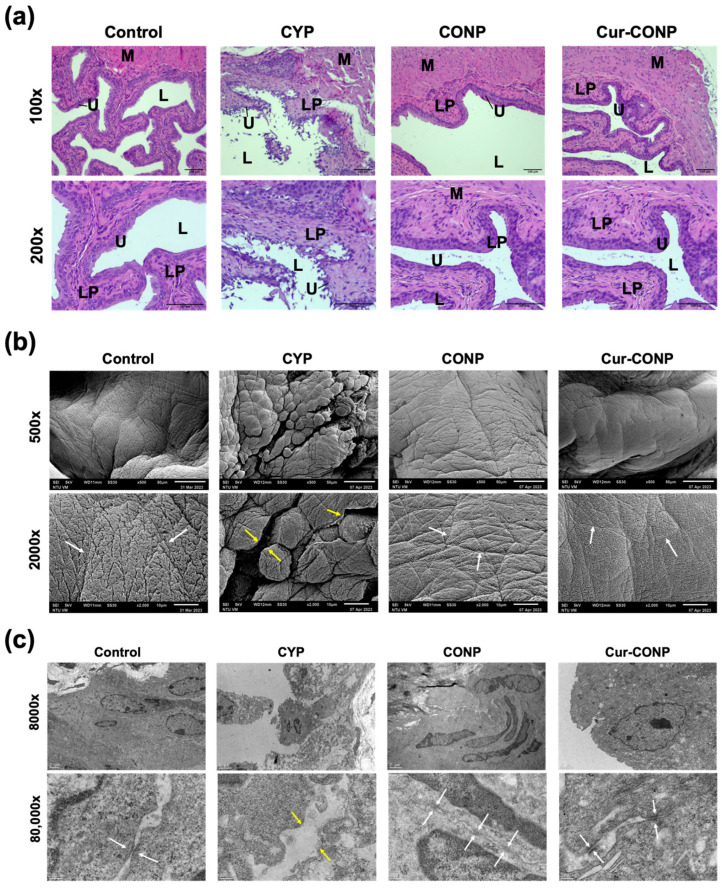
(**a**) The H&E-stained images reveal severe denudation, a thinner urothelium layer, and edema in the CYP group, while the CONP and Cur-CONP groups exhibit intact and thick urothelium layers. Key: U—Urothelium; LP—Lamina propria; M—Smooth muscle; L—Bladder lumen. Scale bar, 100 μm. (**b**) SEM images depicting the bladder walls of the mice. In the CYP group, loose alignment of the urothelium is evident (yellow arrow), while the CONP and Cur-CONP groups exhibit compact urothelium alignments (white arrows). Scale bars for 500× and 2000× magnification are 50 μm and 10 μm, respectively. (**c**) TEM images of the urothelium illustrate compact cell adhesion and cell junctions in the Control group (white arrow). In contrast, the CYP group displays loose urothelium arrangement and disconnections (yellow arrow). The CONP and Cur-CONP groups exhibit compact cell structures and cell junctions similar to those of the Control group. Scale bars for 8000× and 80,000× magnification are 2 μm and 200 nm, respectively.

To assess the bladder tissue morphology, H&E staining was conducted, and the resulting images are presented in Figure 6a. In the CYP group, severe denudation and thinner urothelium layers indicate bladder barrier dysfunction, suggesting a potential invasion of the urinary solutes into the underlying tissues. Conversely, images from the CONP and Cur-CONP groups display intact and thick urothelium layers. SEM analysis of the bladder wall surface reveals loose urothelium alignment in the CYP group (Figure 6b). In contrast, the CONP and Cur-CONP groups exhibit relatively compact urothelium alignments.

Further investigation of the urothelium alignment and morphology at the cellular level was conducted using TEM (Figure 6c). The CYP group exhibits loose cell arrangement and larger spacing between the cells. In comparison, the CONP and Cur-CONP groups demonstrate densely packed urothelium and cell junctions. These results show that CONPs and Cur-CONPs can maintain compact urothelium layers, preventing the invasion of urinary solutes into the underlying tissues.

### 3.5. Western Blot: Expression of Inflammation-Related Proteins

**Figure 7:**
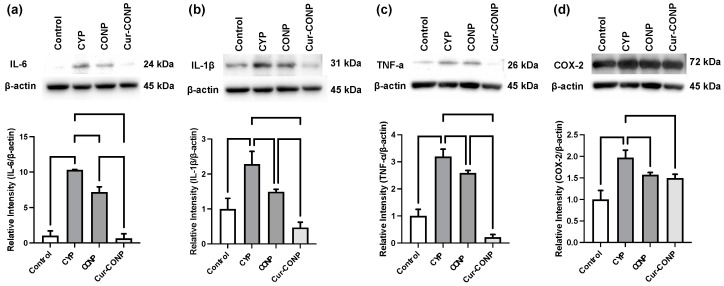
Western blot analysis showing the inflammation-related protein levels of (**a**) *IL-6*, (**b**) *IL-1β*, (**c**) *TNF-α*, and (**d**) *COX-2*. The IC mouse model was induced with CYP. Relative to the CYP group, both the CONP and Cur-CONP groups exhibit significantly lower expression levels of the inflammation-related proteins. Notably, among the protein levels of *IL-6*, *IL-1β*, and *TNF-α*, the Cur-CONP group demonstrates lower inflammatory proteins than the CONP group, indicating superior anti-inflammatory properties.

Western blot analysis was employed to evaluate the inflammation-related proteins (Figure 7). The CYP group exhibited the highest expression of each inflammation-related protein. Comparative analysis revealed that the CONP and Cur-CONP groups demonstrate significantly lower levels of inflammation-related proteins than those in the CYP group. Moreover, among the expression patterns of *IL-6*, *IL-1β*, and *TNF-α*, the Cur-CONP group exhibited even lower levels than the CONP group.

## 4. Discussion

Several studies have consistently highlighted the role of ROS and the impacts of antioxidants in the context of IC. Excess ROS, if not counteracted by antioxidant mechanisms such as superoxide dismutase (SOD) and catalase, can inflict damage on adjacent tissues. This oxidative stress may contribute to bladder barrier dysfunction, resulting in invasion by urinary solutes, the triggering of inflammatory responses, and the manifestation of various IC symptoms. Consequently, our hypothesis posits that ROS play a pivotal role in the pathophysiology of IC, and mitigating excess ROS through scavenging mechanisms could alleviate the symptoms in individuals with IC.

Cerium belongs to the lanthanide group and possesses unique catalytic properties that are attributed to the shielding of the 5p and 4d electrons by the 4f electron orbitals [40]. Furthermore, the oxygen vacancies within the crystal structure contribute to its catalytic capabilities [41]. In contrast to many rare earth elements that exist predominantly in the trivalent state, cerium can exist stably in both the Ce^3+^ and Ce^4+^ states, as shown in Figure 3g,h [42]. The exceptional antioxidant properties of the CONPs stem from their high surface-to-volume ratio, which offers a large reactive surface area. CONPs exhibit SOD-mimetic and catalase-mimetic activities, effectively reducing superoxide and hydrogen peroxide. The binding of superoxide to Ce^3+^ results in its reduction, forming hydrogen peroxide in the process. Moreover, Ce^4+^ acts as a binding site for hydrogen peroxide, undergoing transformation to oxygen while simultaneously experiencing reduction, resulting in the conversion of the binding site from Ce^4+^ to Ce^3+^. Subsequently, Ce returns to its initial state to complete the redox cycle [43]. By reversible switching between the two oxidation states, CONPs present auto-regenerative antioxidant properties for repeatedly scavenging the surrounding ROS. This self-regenerating antioxidant is a valuable characteristic for its potential use as a pharmacological agent. Moreover, with a constant catalytic rate, it presents better activity than SOD [42]. Redox cycling enables the CONPs to serve as long-term antioxidants to treat and control IC without frequent administration compared to conventional treatments.

In our prior study, the administration of CONPs demonstrated significant therapeutic effects on IC by mitigating excessive ROS. Despite their efficacy, CONPs alone were insufficient at effectively alleviating the acute symptoms in IC patients owing to their limited short-term antioxidant properties. Recognizing curcumin’s renowned short-term antioxidant and anti-inflammatory activities, which are attributed to its antioxidant mechanism involving hydrogen abstraction from the phenolic OH group and central CH_2_ group in the heptadienone link [44,45], we attempted to enhance the short-term antioxidant properties by loading curcumin onto CONPs using electrostatic force. The selection of curcumin, despite its known challenges in clinical translation, was based on its extensive preclinical evidence and the innovative approach of using cerium oxide nanoparticles to enhance its bioavailability and efficacy. Curcumin’s unique properties and our preliminary data supporting the efficacy of Cur-CONP justified its selection. Future studies could explore direct comparisons with these other antioxidants to further validate our findings.

After administration of Cur-CONPs, curcumin is released rapidly to swiftly scavenge excess ROS, thereby alleviating acute pain in IC patients. In addition to curcumin, the CONPs exhibit auto-regenerative antioxidant properties, enabling them to scavenge the excess ROS repeatedly. The combination of short-term and long-term antioxidant properties of Cur-CONPs contributes to the elimination of oxidative stress, maintenance of compact urothelium layers, preservation of bladder barrier function, and subsequent reduction in the inflammatory responses and sensory nerve hyperactivity.

Notably, we administered 30 mg/kg CONPs to the CONP group and 30 mg/kg Cur-CONPs to the Cur-CONP group. The Cur-CONP group received 22 mg/kg CONPs and 8 mg/kg curcumin. Despite the higher concentration of CONPs in the CONP group compared to the Cur-CONP group, the Cur-CONP group showed better therapeutic effects on urinary frequency, pelvic pain, and inflammation protein expression. This result demonstrated that curcumin significantly enhances therapeutic outcomes.

This study aimed to evaluate the therapeutic efficacy of Cur-CONPs in alleviating the symptoms associated with IC. We examined the antioxidant and anti-inflammatory properties as well as their impact on relieving urinary frequency and pelvic pain.

In this study, we successfully synthesized Cur-CONPs through a green method, which presented well-crystalized homogeneous dispersion and good biocompatibility (Figure S4). The particles showed a more negative zeta potential after loading with curcumin (Figure 3d,e). It is known that a higher magnitude of zeta potential indicates a stronger electrostatic force between the particles; therefore, the zeta potential serves as an indicator of colloidal stability, including the tendency for aggregation [46,47]. Analysis of the size distribution revealed that Cur-CONPs exhibit a narrower distribution than CONPs (Figure 3f), signifying improved colloidal stability achieved by loading curcumin on the CONPs. The interconnected aspects of the zeta potential and size distribution allow a comprehensive assessment of the colloidal stability, which is vital for understanding the behaviors of nanoparticles in solution. Surprisingly, XPS analysis revealed that loading curcumin on CONPs increased the Ce^3+^/Ce^4+^ ratio (Figure 3g,h). This higher ratio signifies more oxygen vacancies, facilitating easier oxygen exchange and redox reactions, particularly SOD-mimetic activities [38,39]. That is, Cur-CONPs may exhibit superior SOD-mimetic antioxidant properties than CONPs.

Among all the symptoms of IC patients, frequent urination and pelvic pain are mainly responsible for decreasing the quality of life and contributing to concomitant anxiety as well as depression. Generally, the urinary frequency of an IC patient can be up to 50 times a day [48]. We assessed the therapeutic impacts of Cur-CONPs on the urinary behaviors and pelvic pain sensitivity (Figure 5).

In the Void Spot assay, assessing the total void volume eliminated the potential void volume differences among groups, ensuring accurate void spot number evaluations. This common practice in urinary studies enhances the reliability of the results. Despite having similar total void volumes, the CYP group exhibited a higher urinary frequency (Figure 5c), while the Cur-CONP group demonstrated a lower urinary frequency, indicating its therapeutic effects on IC-induced urinary frequency.

Pelvic pain evaluations conducted through the mechanical withdrawal threshold and response rate (Figure 5d,e) reveal reduced pain sensitivity and response frequency in the Cur-CONP group compared to the CYP group. Overall, the behavioral studies demonstrated significant reductions in urinary frequency and pelvic pain sensitivity in mice with IC.

The proposed hypothesis that preserving the bladder barrier function by reducing excess ROS could ameliorate IC symptoms was validated by histological analysis of the bladder tissue (Figure 6). Compared to the CYP group, IC mice treated with Cur-CONPs displayed compact and thickened urothelium layers. TEM images further revealed the presence of cell junctions in the Cur-CONP group, indicating potential integrity in the urothelium layers and maintenance of the bladder barrier function. Future studies should therefore explore the expression of cell junction proteins to enhance this understanding.

Invading urinary solutes due to bladder barrier dysfunction can initiate inflammatory responses, potentially leading to chronic inflammation. In turn, inflammation triggers sensory afferent nerve upregulation, bladder afferent sensory nerve hyperactivity, and neuroplasticity [49]. Lowered thresholds for the nociceptive and mechanoreceptive responses contribute to heightened pain sensations and increased urinary frequency, as even a reduced bladder volume can activate the nerves [50]. Therefore, we evaluated the inflammation proteins in this study, as shown in Figure 7. The results demonstrate that Cur-CONPs mitigate the inflammatory responses in IC mice, which is consistent with our in vitro RT-qPCR finding. Connecting these results with those from previous behavioral studies and histological evaluations, the antioxidant and anti-inflammatory properties of Cur-CONPs appear integral to maintaining the bladder barrier function, reducing urinary frequency, and pain sensitivity.

Our mouse study provides valuable insights into the novel treatment proposed for IC. While these findings contribute significantly to our understanding, it is essential to consider their translational implications for human health. Although IC in humans is known for its complexity and heterogeneity, the CYP-induced mouse model of IC can mimic certain key aspects of IC, including oxidative stress, inflammation, urothelial damage, and bladder dysfunction. This model enables us to study the therapeutic effects of Cur-CONPs through their antioxidant mechanisms. In conclusion, while our mouse study serves as a foundational step, further investigation in human populations is warranted to fully elucidate the translational potential of our findings. Hence, this study opens further avenues for collaborative and interdisciplinary research that can ultimately benefit human health.

## 5. Conclusions

Oxidative stress is considered the etiology of IC, making antioxidants a promising strategy for improving the symptoms. In this study, we designed Cur-CONPs with full-spectrum antioxidant properties to alleviate the symptoms of IC. Loading curcumin on CONPs enhanced the colloidal stability and anti-inflammatory properties. In vivo studies were performed to validate the therapeutic effects on urinary frequency and pelvic pain reduction. Cur-CONPs effectively scavenged ROS, preserving a functional bladder barrier, as confirmed by the histological evaluation, and maintaining compact urothelium alignment and cell junction structure. Western blot results demonstrated that Cur-CONPs could decrease inflammation-related protein expressions, indicating their anti-inflammatory properties at the protein level. In summary, Cur-CONPs exhibit robust therapeutic effects with promising results for relieving the symptoms of IC.

## Figures and Tables

**Table 1:** Primer sequences for RT-qPCR.

Gene	Accession Number		Sequence (5′-3′)
*GAPDH*	NM_002046	Forward	AAGGTGAAGGTCGGAGTCAAC
		Reverse	GGGGTCATTGATGGCAACAATA
*IL-6*	NM_000600	Forward	ACTCACCTCTTCAGAACGAATTG
		Reverse	CCATCTTTGGAAGGTTCAGGTTG
*IL-1β*	NM_000576	Forward	GAAATGCCACCTTTTGACAGTG
		Reverse	TGGATGCTCTCATCAGGACAG
*TNF-α*	NM_000594	Forward	CCCGAGTGACAAGCCTGTAG
		Reverse	GATGGCAGAGAGGAGGTTGAC
*COX-2*	NM_000963	Forward	GTTGCTGGGGGAAGGAATGT
		Reverse	AGCATCTGGACGAGGCTTTT

## Data Availability

Data is contained within the article.

## Supplementary Materials

The following supporting information can be downloaded at: https://www.mdpi.com/article/10.3390/antiox13070826/s1. Figure S1. (a) SEM images of CONP showed no significant aggregation. (b) TEM high-resolution images of CONP showed the interplanar spacing of the lattice plane (111). Scale bar, 2 nm. (c) TEM electron diffraction pattern of CONP displayed the lattice planes (111), (002), (022), (113), and (222). Figure S2. (a) EDX spectrum of Cur-CONP. (b) TEM high-resolution images; (c) Elemental mapping for oxygen. (d) Elemental mapping for cerium. Results confirm oxygen and cerium aggregation, verifying the presence of cerium oxide. Curcumin characterization requires further study. Figure S3. The standard curve of curcumin. Figure S4. Biocompatibility test. (a) Live/Dead assay results revealed minimal cell toxicity in CONP and Cur-CONP groups compared to the Positive group. (b) WST-1 assay demonstrated cell viability exceeding 70%, indicating low cytotoxicity for Cur-CONP. Figure S5. Liver H&E staining images. (a) The Control group displayed well-preserved cytoplasm with no pathological changes. Similarly, images of (b) CYP, (c) CONP, and (d) Cur-CONP groups showed no significant difference compared to the Control group. Thus, CONP and Cur-CONP demonstrated minimal hepatic toxicity. Figure S6. Kidney H&E staining images. (a) The Control group exhibited well-preserved cytoplasm with no pathological changes. Similarly, images of (b) CYP, (c) CONP, and (d) Cur-CONP groups showed no significant difference compared to the Control group. Hence, CONP and Cur-CONP demonstrated minimal renal toxicity. Figure S7. Full, unedited gel for *IL-6* (Figure 7a), and *IL-1β* (Figure 7b). Rectangles highlight specific regions within each image, indicating where cropped images were extracted. Figure S8. Full, unedited gel for *β-actin* (Figure 7a,b). Rectangles highlight specific regions within each image, indicating where cropped images were extracted. Figure S9. Full unedited gel for *TNF-α* (Figure 7c). Rectangles highlight specific regions within each image, indicating where cropped images were extracted. Figure S10. Full unedited gel for *COX-2* (Figure 7d) and β-actin (Figure 7c,d). Rectangles highlight specific regions within each image, indicating where cropped images were extracted. Table S1. The XPS characteristic peaks of CONP and Cur-CONP. Table S2. Serum chemical analysis. Concentrations of Alanine aminotransferase (ALT) and blood urea nitrogen (BUN) demonstrated that Cur-CONP exhibited minimal hepatic and renal toxicity.
