# Supplementary material for: Characterization and Therapeutic Potential of Curcumin-Loaded Cerium Oxide Nanoparticles for Interstitial Cystitis Management
Source: Antioxidants (Basel). 2024 Jul 10;13(7):826. doi: 10.3390/antiox13070826 (PMC11273629; doi:10.3390/antiox13070826)
Supplement: Supplementary file 1 [file antioxidants-13-00826-s001.zip › antioxidants-3063897-supplementary.pdf]

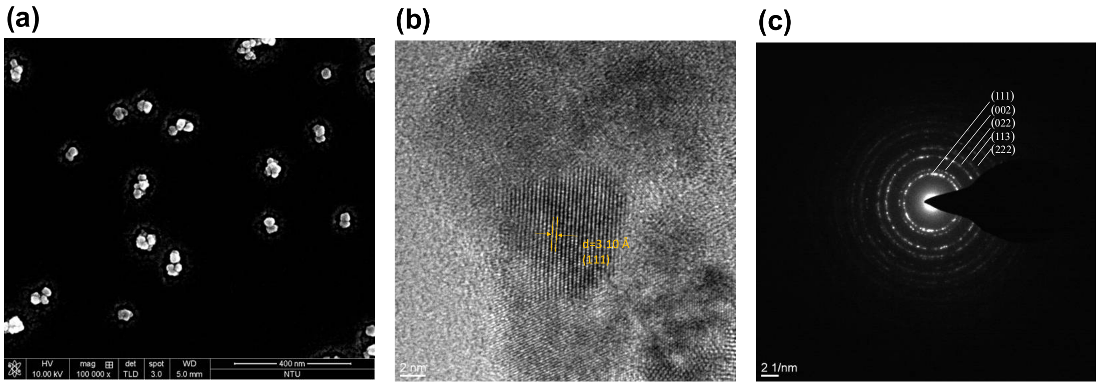

Figure S1 (a) SEM images of CONP showed no significant aggregation. (b) TEM high-resolution images of CONP showed the interplanar spacing of the lattice plane (111). Scale bar, 2 nm. (c) TEM electron diffraction pattern of CONP displayed the lattice planes (111), (002), (022), (113), and (222).

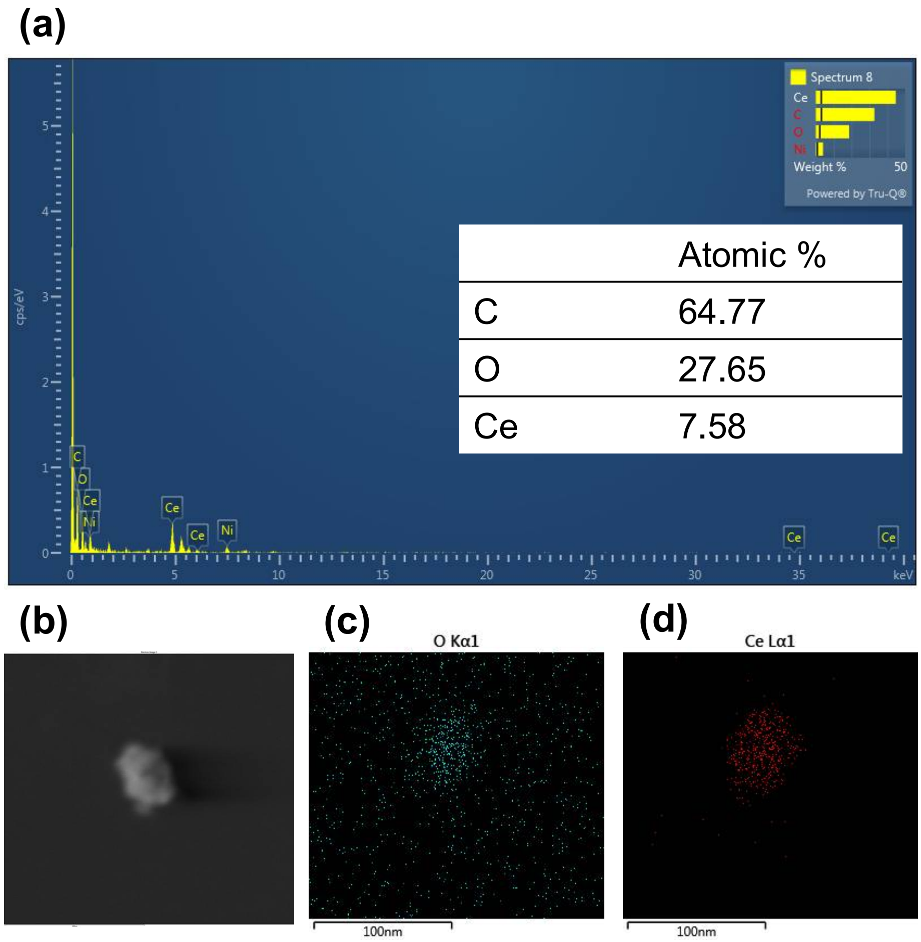

Figure S2 (a) EDX spectrum of Cur-CONP. (b) TEM high-resolution images; (c) Elemental mapping for oxygen. (d) Elemental mapping for cerium. Results confirm oxygen and cerium aggregation, verifying the presence of cerium oxide. Curcumin characterization requires further study.

Table S1 The XPS characteristic peaks of CONP and Cur-CONP

	Reference peak position (eV)	Peak position (eV)	
		CONP	Cur-CONP
$Ce^{3+}3d_{3/2}$	903.4	903.2	905.3
	898	898.1	898.1
$Ce^{3+}3d_{5/2}$	884.5	884.8	885.5
	880.9	880.5	880.8
$Ce^{4+}3d_{3/2}$	916.3	916.0	916.0
	907.0	906.9	911.2
	900.7	900.9	900.6
$Ce^{4+}3d_{5/2}$	898.3	897.1	896.9
	888.5	888.3	889.0
	882.0	882.1	882.3

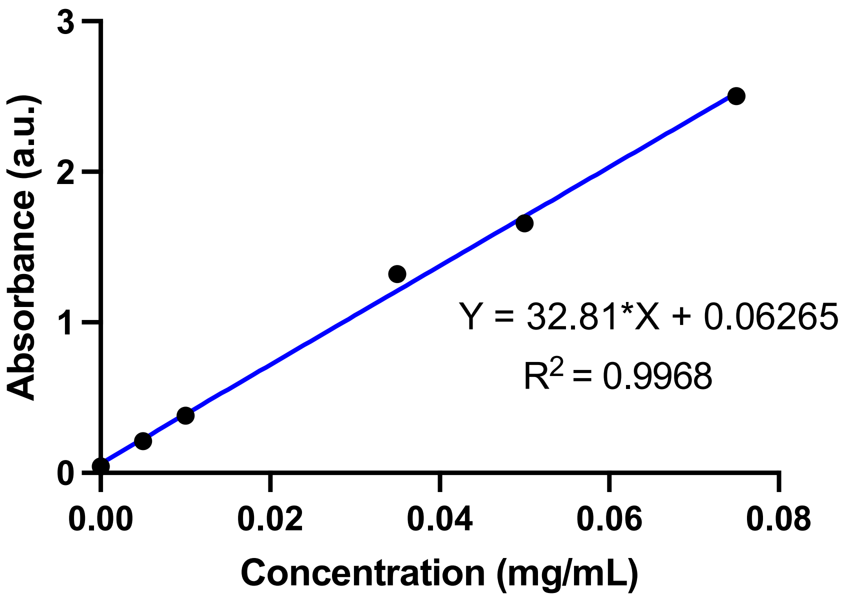

Figure S3 The standard curve of curcumin.

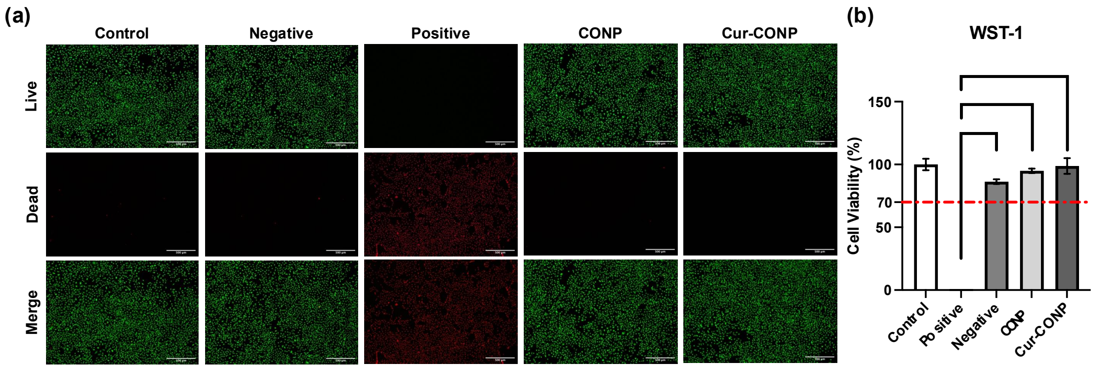

Figure S4 Biocompatibility test. (a) Live/Dead assay results revealed minimal cell toxicity in CONP and Cur-CONP groups compared to the Positive group. The Scale bars are 500 µm. (b) WST-1 assay demonstrated cell viability exceeding 70%, indicating low cytotoxicity for Cur-CONP. ISO-10993-5 considers cell viability below 70% as indicative of cytotoxic potential. The results affirm the low cytotoxicity of Cur-CONP.

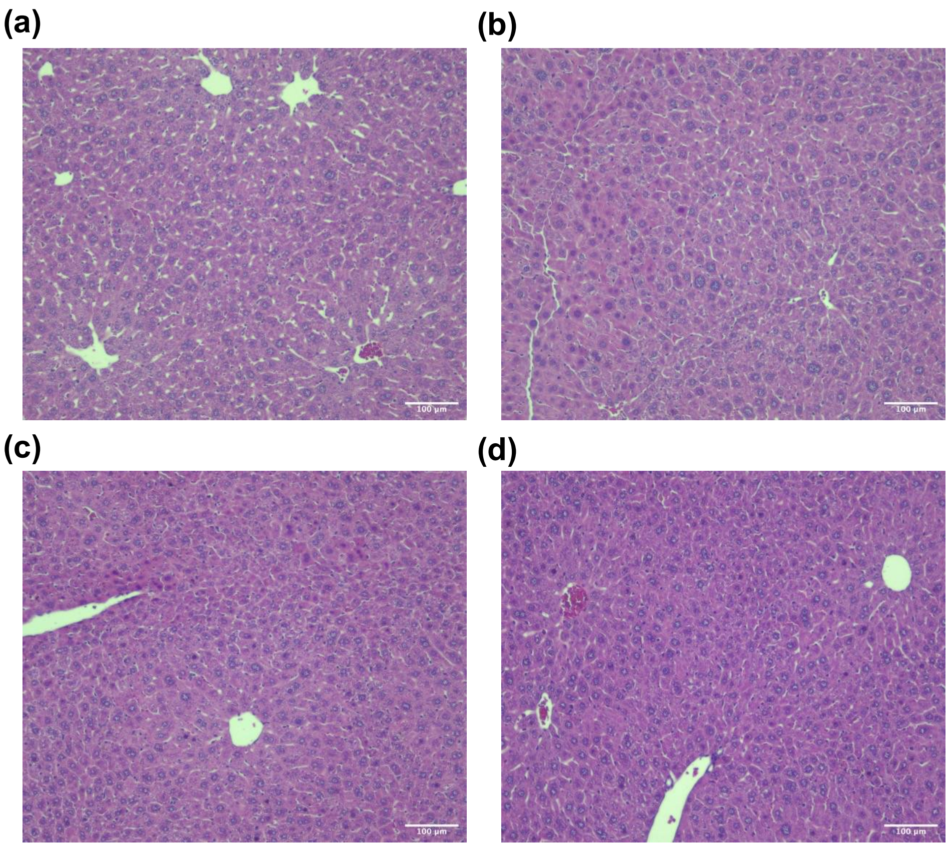

Figure S5 Liver H&E staining images. (a) The Control group displayed well-preserved cytoplasm with no pathological changes. Similarly, images of (b) CYP, (c) CONP, and (d) Cur-CONP groups showed no significant difference compared to the Control group. Thus, CONP and Cur-CONP demonstrated minimal hepatic toxicity.

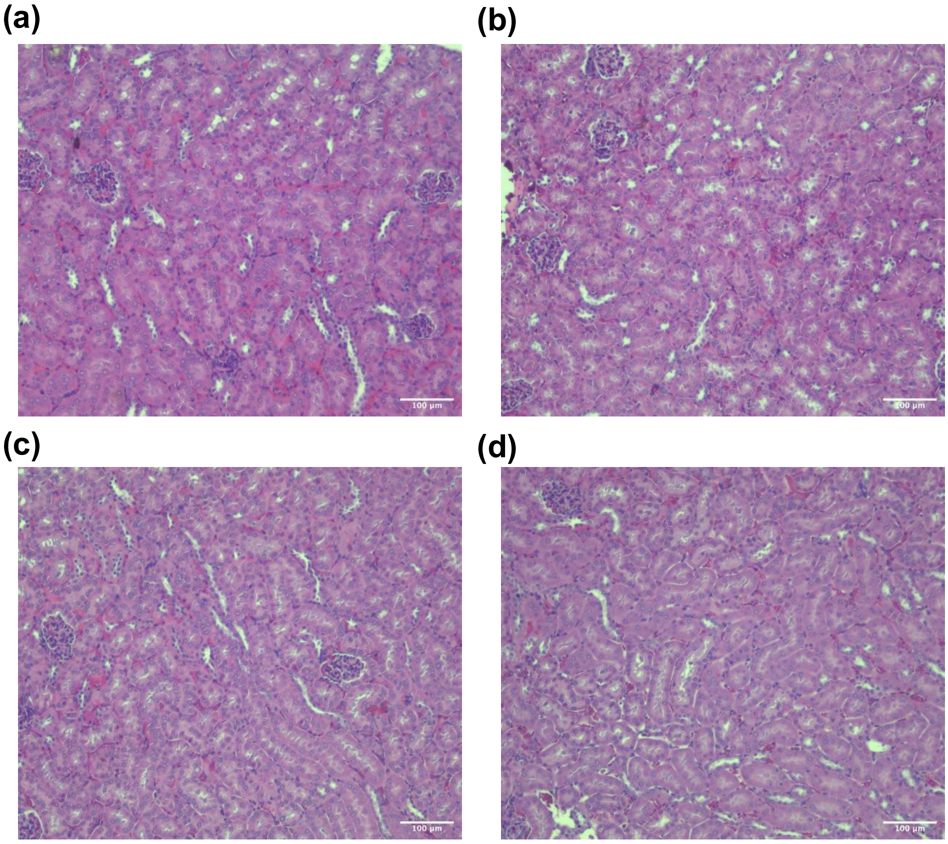

Figure S6. Kidney H&E staining images. (a) The Control group exhibited well-preserved cytoplasm with no pathological changes. Similarly, images of (b) CYP, (c) CONP, and (d) Cur-CONP groups showed no significant difference compared to the Control group. Hence, CONP and Cur-CONP demonstrated minimal renal toxicity.

Table S2. Serum chemical analysis. Concentrations of Alanine aminotransferase (ALT) and blood urea

nitrogen (BUN) demonstrated that Cur-CONP exhibited minimal hepatic and renal toxicity.

	Control	CYP	Cur-CONP
ALT (U/L)	36±2.65	29±1.53	29±1.73
BUN (mg/dL)	24.73±1.69	22.78±0.94	19.75±0.78

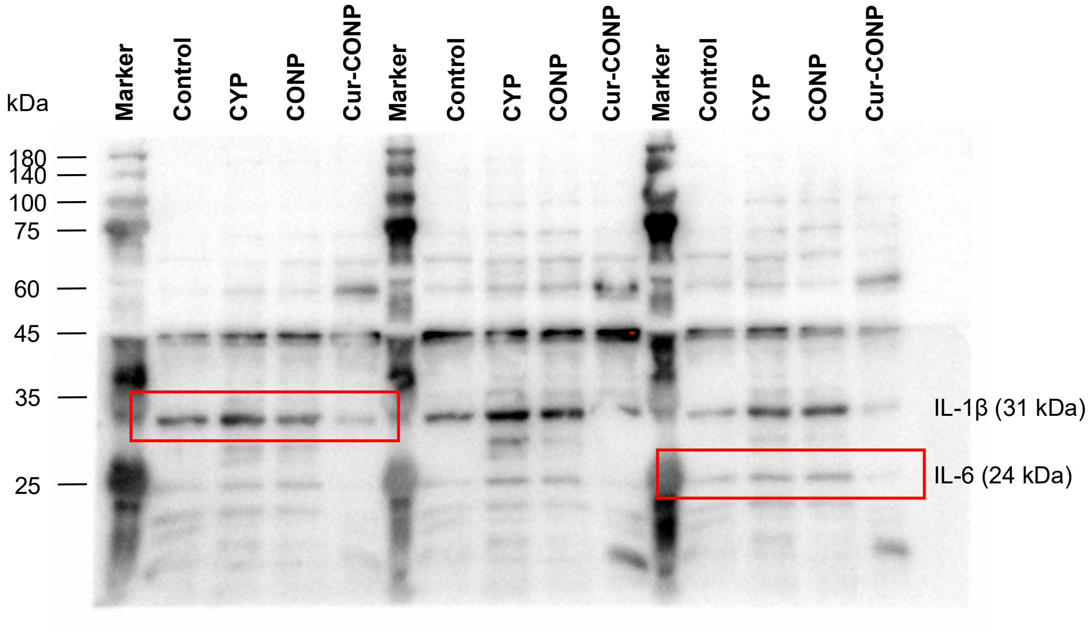

Figure S7. Full, unedited gel for *IL-6* (Figure 7(a)) and IL-1β (Figure 7(b)). Rectangles highlight specific regions within each image, indicating where cropped images were extracted.

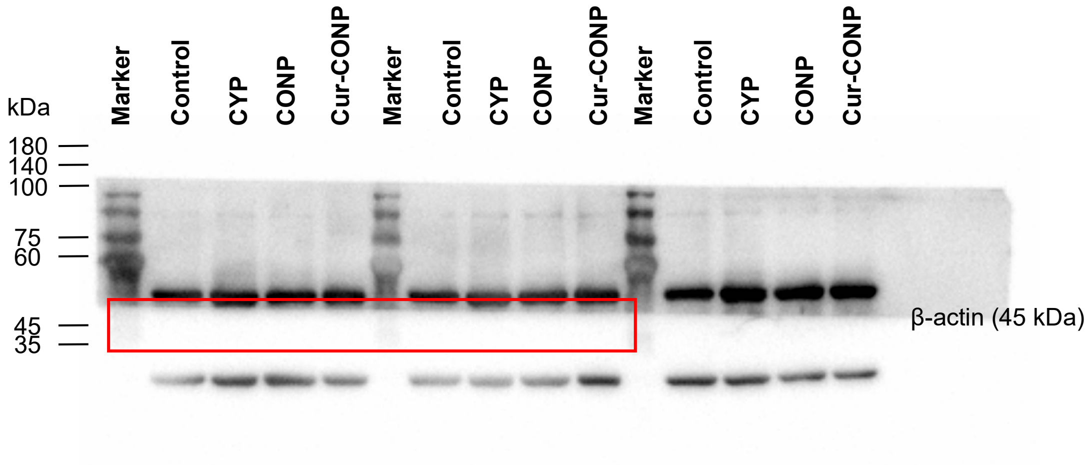

Figure S8. Full, unedited gel for *β-actin* (Figure 7(a) and Figure 7(b)). Rectangles highlight specific regions within each image, indicating where cropped images were extracted.

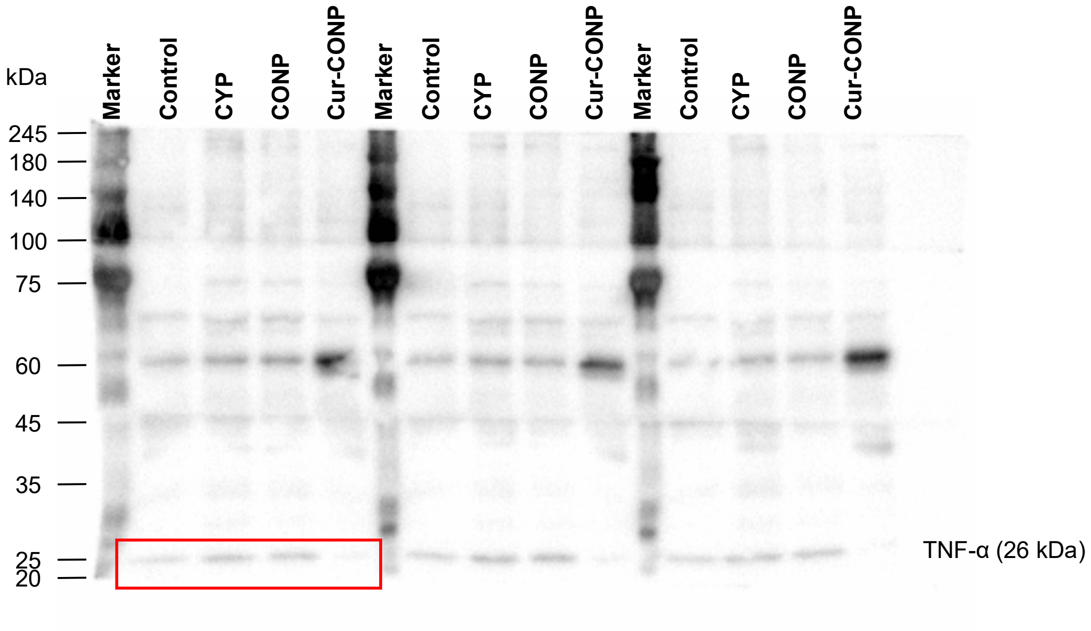

Figure S9. Full unedited gel for *TNF-α* (Figure 7(c)). Rectangles highlight specific regions within each image, indicating where cropped images were extracted.

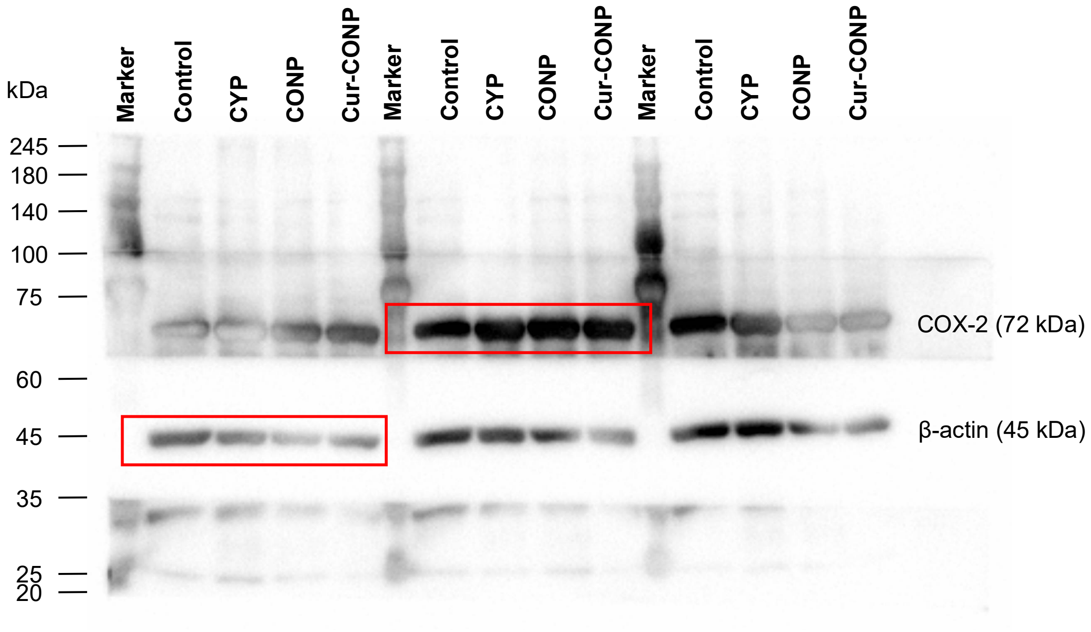

Figure S10. Full unedited gel for *COX-2* (Figure 7(d)) and *β-actin* (Figure 7(c) and Figure 7(d)). Rectangles highlight specific regions within each image, indicating where cropped images were extracted.
